# Genomic diversity of class I integrons from antimicrobial resistant strains of *Salmonella* Typhimurium isolated from livestock, poultry and humans

**DOI:** 10.1371/journal.pone.0243477

**Published:** 2020-12-11

**Authors:** Sangeeta Rao, Lyndsey Linke, Enrique Doster, Doreene Hyatt, Brandy A. Burgess, Roberta Magnuson, Kristy L. Pabilonia, Paul S. Morley

**Affiliations:** 1 Department of Clinical Sciences, College of Veterinary Medicine and Biomedical Sciences, Colorado State University, CO, United States of America; 2 Department of Veterinary Population Medicine, College of Veterinary Medicine, University of Minnesota, MN, United States of America; 3 Veterinary Education, Research, and Outreach Program, Texas A&M University and West Texas A&M University, College Station, TX, United States of America; 4 Department of Microbiology, Immunology and Pathology, College of Veterinary Medicine and Biomedical Sciences, Colorado State University, Fort Collins, CO, United States of America; 5 College of Veterinary Medicine, University of Georgia, Athens, GA, United States of America; Nitte University, INDIA

## Abstract

**Introduction:**

Multidrug resistance (MDR) is a serious issue prevalent in various agriculture-related foodborne pathogens including *Salmonella enterica* (*S*. *enterica*) Typhimurium. Class I integrons have been detected in *Salmonella* spp. strains isolated from food producing animals and humans and likely play a critical role in transmitting antimicrobial resistance within and between livestock and human populations.

**Objective:**

The main objective of our study was to characterize class I integron presence to identify possible integron diversity among and between antimicrobial resistant *Salmonella* Typhimurium isolates from various host species, including humans, cattle, swine, and poultry.

**Methods:**

An association between integron presence with multidrug resistance was evaluated. One hundred and eighty-three *S*. Typhimurium isolates were tested for antimicrobial resistance (AMR). Class I integrons were detected and sequenced. Similarity of AMR patterns between host species was also studied within each integron type.

**Results:**

One hundred seventy-four (95.1%) of 183 *S*.Typhimurium isolates were resistant to at least one antimicrobial and 82 (44.8%) were resistant to 5 or more antimicrobials. The majority of isolates resistant to at least one antimicrobial was from humans (45.9%), followed by swine (19.1%) and then bovine (16.9%) isolates; poultry showed the lowest number (13.1%) of resistant isolates. Our study has demonstrated high occurrence of class I integrons in *S*. Typhimurium across different host species. Only one integron size was detected in poultry isolates. There was a significant association between integron presence of any size and specific multidrug resistance pattern among the isolates from human, bovine and swine.

**Conclusions:**

Our study has demonstrated a high occurrence of class I integrons of different sizes in *Salmonella* Typhimurium across various host species and their association with multidrug resistance. This demonstration indicates that multidrug resistant *Salmonella* Typhimurium is of significant public health occurrence and reflects on the importance of judicious use of antimicrobials among livestock and poultry.

## Introduction

Emerging and existing antimicrobial drug resistance (AMR) including multidrug resistance (MDR) in bacteria is a major public health concern with global relevance to overall human and animal health [1]. An early screening solution to identify AMR is an essential step to effectively manage disease spread and reduce the number of new cases in humans and livestock.

*Salmonella enterica* bacteria can be associated with health and production issues for livestock and poultry. *Salmonella enterica* subsp. *enterica* serotype Typhimurium (*S*. Typhimurium) is a globally recognized human pathogen and poses a food safety risk [2–4]. It can infect a wide range of hosts including animals such as poultry, pigs, sheep and cattle [5]. *S*. Typhimurium ranks among the top five serotypes recovered from food production animals [2,6], and is one of the primary causes of human foodborne infections and outbreaks in many countries [4,7,8].

Multidrug resistance (MDR) is a serious issue prevalent in various agriculture-related foodborne pathogens including *S*. *enterica*. Presence of AMR in *Salmonella* has been well documented for many years, and isolation of MDR (resistant to 3 or more antimicrobial classes) [9,10]. *S*. Typhimurium isolates have been increasing since the mid-1960s [11]. Even as early as 1994, 62% of isolates were multi-drug resistant [12]. Zhao et al (2005) [2] have demonstrated that 76% of *S*. Typhimurium isolated from cattle, chickens, pigs, turkeys and their meats, and from companion animals were resistant to at least 1 antimicrobial.

Antimicrobial resistances in *Salmonella* has been associated with the presence of integrons [13–15]. Integrons are bacterial genetic elements that allow the shuffling of smaller mobile elements called gene cassettes [16] and are horizontally transmissible. Integrons were constituents of the first resistance plasmids reported, conferring resistance to aminoglycosides, chloramphenicol and sulphonamides [16]. Class I integrons can incorporate AMR genes from the environment by site-specific recombination [17]. Of the 5 known classes of integrons, class I integrons are the most prevalent and have been detected in up to 71% of fecal samples from lot-fed cattle [18], and in 22% to 59% of Gram-negative human clinical isolates [19,20]. Thirty-nine percent of isolates were reported to contain class I integrons from meat and dairy products [15], 25.6% from poultry and swine [21], 46% from swine environment [14], 34% and 75% from swine and human isolates [22] and 51% from various animals and their meat products [2]. As such, integrons likely play a critical role in transmitting AMR within and between livestock and human populations.

In a swine study, Rao et al (2008) [14] demonstrated a strong association between specific class I integrons of 3 sizes (1,000, 1,200 and 1,600bp) and AMR among 730 *Salmonella enterica* isolates. The most common MDR pattern identified in *S*. *enterica* included ampicillin, chloramphenicol, streptomycin, sulfisoxazole and tetracycline (ACSSuT), 99% of which carried a specific class I integron. The association between presence of integrons and AMR patterns has also been found by other researchers [23,24].

The goal of this study was to characterize class I integron diversity between and among *S*. Typhimurium isolates from various host species, including humans, cattle, swine, and poultry. The specific aim was to evaluate the association of integrons with MDR in S. Typhimurium within livestock, poultry and human species. If such an association exists, screening assays could be developed that would allow early MDR detection and improved treatment of current and emerging drug resistant *S*. Typhimurium in humans and animals.

## Materials and methods

### Selection of *Salmonella* Typhimurium *isolates*

Multiple laboratories across the United States were contacted for *Salmonella* Typhimurium repositories from human, bovine, swine and poultry. The isolates were chosen from six different and independent institutes across the United States as follows: Human isolates collected between 2009–2014 from two institutes, Bovine isolates collected between 2009–2012 and poultry isolates collected between 2009–2013 from two institutes, Porcine isolates collected between 2009–2013 from three institutes (one represents the same institute as for bovine and poultry isolates). The isolates were selected from different time points, spanning a five year time period. They were not replicates of the same isolate and were randomly selected from the database provided by our collaborators. One hundred and eighty-three isolates were shipped to our laboratory: 88 human, 33 bovine, 36 swine and 26 poultry isolates. Briefly, samples were streaked for isolation onto trypticase soy agar plates (Becton, Dickinson and Company, Franklin Lakes, NJ) containing 5% sheep blood and incubated overnight at 37°C. After verification as serogroup B *Salmonella* (BD Diagnostic Systems®, Becton, Dickinson and Company) by traditional slide agglutination, an isolated colony was inoculated into 1mL of trypticase soy broth and incubated overnight at room temperature. After mixing in sterile glycerol to a final glycerol concentration of 10%, the cultures were frozen at -80°C until they were retrieved for further testing.

### Antimicrobial susceptibility testing

All *Salmonella* Typhimurium isolates, independent of integron presence, were tested for susceptibility to 16 antimicrobial agents by the Kirby-Bauer agar disk diffusion assay [25]. The antimicrobial drugs and their potencies were: amoxicillin-clavulanate 20/10μg (AMC); ampicillin 10μg (AM); chloramphenicol 30μg (C); cephalothin 30μg (CF); ceftiofur 30μg (CTO); enrofloxacin 5μg (ERF); streptomycin 10μg (S); sulfisoxazole 250μg (SSS); tetracycline 30μg (TE); trimethoprim-sulfamethoxazole 1.25/23.75μg (SXT); cefoxitin 30μg (FOX); ciprofloxacin 5μg (CIP); florfenicol μg (FFC); gentamicin 10μg (GM); kanamycin 30μg (K); and nalidixic acid 30μg (NA). *Escherichia coli* ATCC 25922 and *Staphylococcus aureus* ATCC 25923 were utilized as quality control organisms.

### Molecular identification of integrons

Primers that correspond to the 5’ conserved segment (CS) and 3’CS portion of class I integrons were utilized to amplify any AMR genes within [14,26]. The primer sequences were forward: 5’-GGC ATC CAA GCA GCA AGC-3’, and reverse: 5’-AAG CAG ACT TGA CCT GAT-3’ [14,27].The isolates underwent the PCR protocols and thermocycler conditions mentioned in Rao et al., 2008 [14] to identify integron sizes. Two positive control samples for class I integrons with sizes of 1,000, 1,200, and 1,600bp were included (5 pg total) with each PCR [14]. PCR products of the isolates with integrons were separated on a 1% agarose gel containing markers to validate band sizes and integron bands were subsequently excised for purification and sequencing.

### Genetic sequencing of integrons

DNA purification of the excised integrons was performed using the QIAquick PCR Purification kit (Qiagen®), followed by evaluation for sample quality, purity and concentration utilizing spectrophotometry, and purified DNA was then sequenced. Briefly, samples were prepared using ABI’s BigDye® Terminator v3.1 sequencing chemistry and processed using ABI 3130xL Genetic Analyzer (Applied Biosystems™, Thermo Fisher). Sequences were generated using both the forward and reverse primers for a complete annotation of each integron sequence.

### Data analysis

Descriptive statistics were performed using frequencies and a heat map was developed to represent the AMR patterns among host species within each integron type. The AMR results were represented as susceptible, intermediate and resistant on the heat map. For statistical analysis, intermediate results were considered resistant. A Fisher’s exact test or a Chi-square analysis was performed to evaluate the association between integron size and certain MDR patterns using SAS v9.4 (SAS Institute Inc., Cary, NC).

### Analysis of integron sequences

Each integron sequence (forward and reverse) was converted to FASTA format and merged using De Novo assembly, followed by alignment with MEGARes, a comprehensive database of antimicrobial resistance determinants [28], for identification of AMR genes. The genes with highest query coverage and % pairwise identity were identified, along with the gene class. The AMR genes within each integron across various host species were represented using a dendrogram, that was created to elucidate the genetic relatedness among integrons using a cut-off of 90% similarity (UPGMA: unweighted pair group with arithmetic mean method) [29]. All analyses were performed with Geneious® 10.2.5 (Biomatters Limited) [30].

## Results

### Antimicrobial resistance patterns

One hundred seventy-four (95.1%) of 183 *S*.Typhimurium isolates were resistant to at least one antimicrobial and 82/183 (44.8%) were resistant to 5 or more antimicrobials.

The percentage of isolates resistant to at least one antimicrobial were: 45.9% of human isolates, 19.1% of swine, 16.9% of bovine and 13.1% of poultry isolates. The highest number of resistances was observed towards streptomycin, followed by tetracycline and sulfisoxazole among all four hosts in the study (Table 1).

**Table 1:** Number and percentage of isolates exhibiting antimicrobial resistance by host species.

Host	# isolates		Amp	C	Str	Sul	Tet	Flor	Am-Cl	Tio	Fox	Ceph	Tri-Sul	Enro	Cip	Gen	Kan	NA
**Human**	**88**	**n**	34	19	81	46	57	19	23	11	10	16	10	9	-	13	7	16
		**%**	38.6	21.6	92	52.3	64.8	21.6	26.1	12.5	11.4	18.2	11.4	10.2	-	14.8	8	18.2
**Bovine**	**33**	**n**	22	20	30	23	26	20	21	10	10	10	1	-	-	5	2	
	** **	**%**	66.7	60.6	90.9	69.7	78.8	60.6	63.6	30.3	30.3	30.3	3	-	-	15.2	6.1	0
**Swine**	**36**	**n**	27	26	33	31	34	26	25	-	5	5	5	6	-	-	-	5
	** **	**%**	75	72.2	91.7	86.1	94.4	72.2	69.4	-	13.9	13.9	13.9	16.7	-	-	-	13.9
**Poultry**	**26**	**n**	2	-	21	7	15	1	-	-	-	-	1	-	1	-	1	-
		**%**	7.7	-	80.8	26.9	57.7	3.8	-	-	-	-	3.8	-	3.8	-	3.8	-
**Total**	**183**		**85**	**65**	**165**	**107**	**132**	**66**	**69**	**21**	**25**	**31**	**17**	**15**	**1**	**18**	**10**	**21**

Amp: Ampicillin; C: Chloramphenicol; Str: Streptomycin; Sul: Sulfisoxazole; Tet: Tetracycline; Flor: Florfenicol; Am-Cl: Amoxicillin-Clavulanate; Tio: Ceftiofur; Fox: Cefoxitin; Ceph: Cephalothin; Tri-Sul: Trimethoprim-sulfa; Enro: Enrofloxacin; Cip: Ciprofloxacin; Gen: Gentamicin; Kan: Kanamycin; NA: Nalidixic acid.

### Integron identification

Forty-two percent (77/183) of the 183 isolates carried at least one class I integron, represented most by swine (16.4%); followed by humans (15.3%), bovine (8.7%) and poultry (1.6%). All of the isolates (100%) carrying any size integron were resistant to at least one antimicrobial. Of all resistant samples, 100% of the integron-carrying bovine isolates and 78.6% of the integron-carrying human isolates were resistant to at least 5 antimicrobials. There were 9 isolates (9/183) that did not show any AMR towards any of the tested drugs and all 9 did not carry any class I integrons.

**Fig 1:**
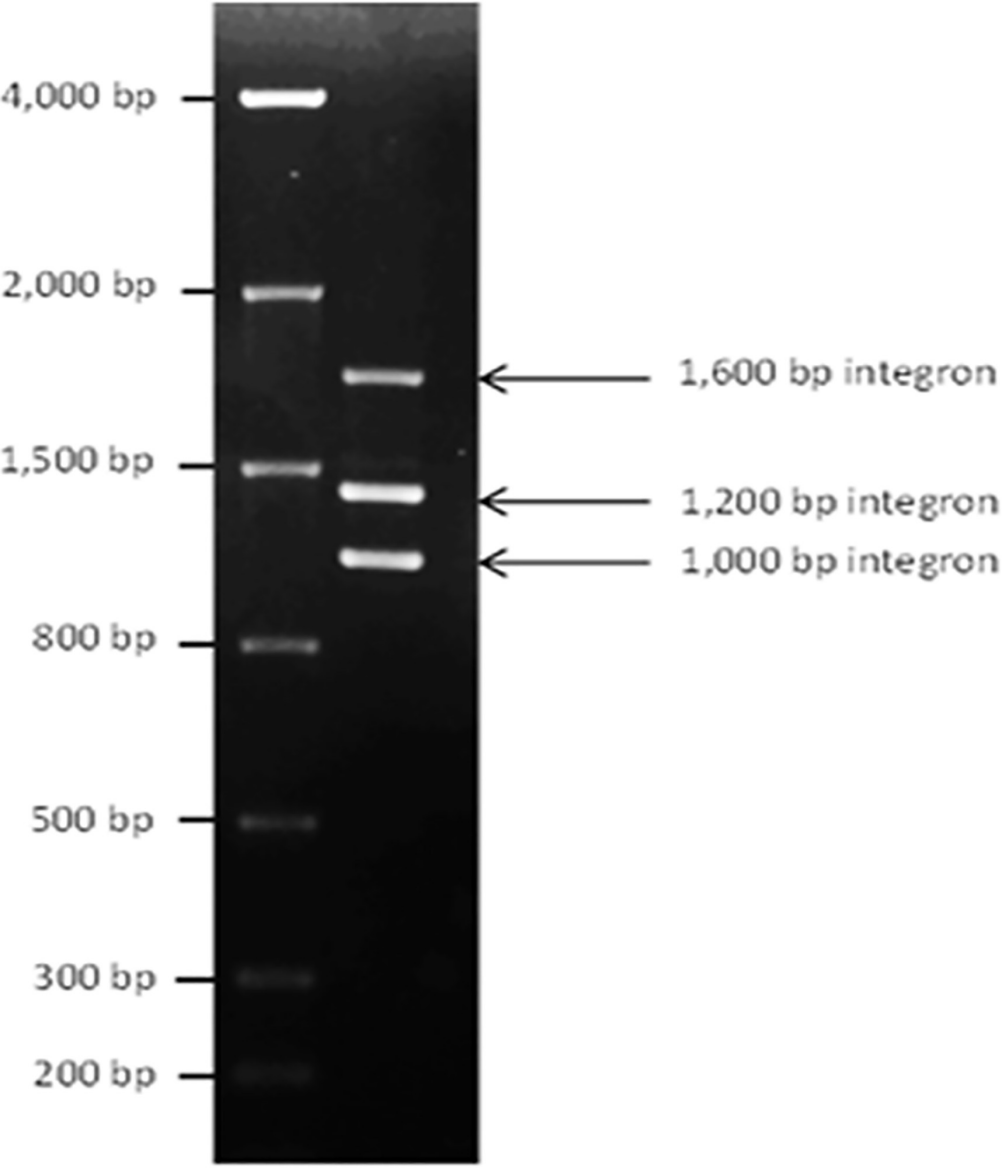
Gel electrophoresis showing sizes of integrons (bp).

The molecular size of integrons that were found in bovine, swine, poultry, and human isolates were characterized (Fig 1). Isolates collected from humans showed the highest variety of integron sizes, which included 1000bp, 1200bp, 1800bp, 2500bp, a combination of 1000bp and 1200bp, and a combination of 1000bp, 1200bp and 1600bp.

Swine and bovine isolates contained 1000bp, the 1800bp integron and a combination of 1000bp and 1200bp integrons. The only integron size detected in poultry was 1000bp.

**Fig 2:**
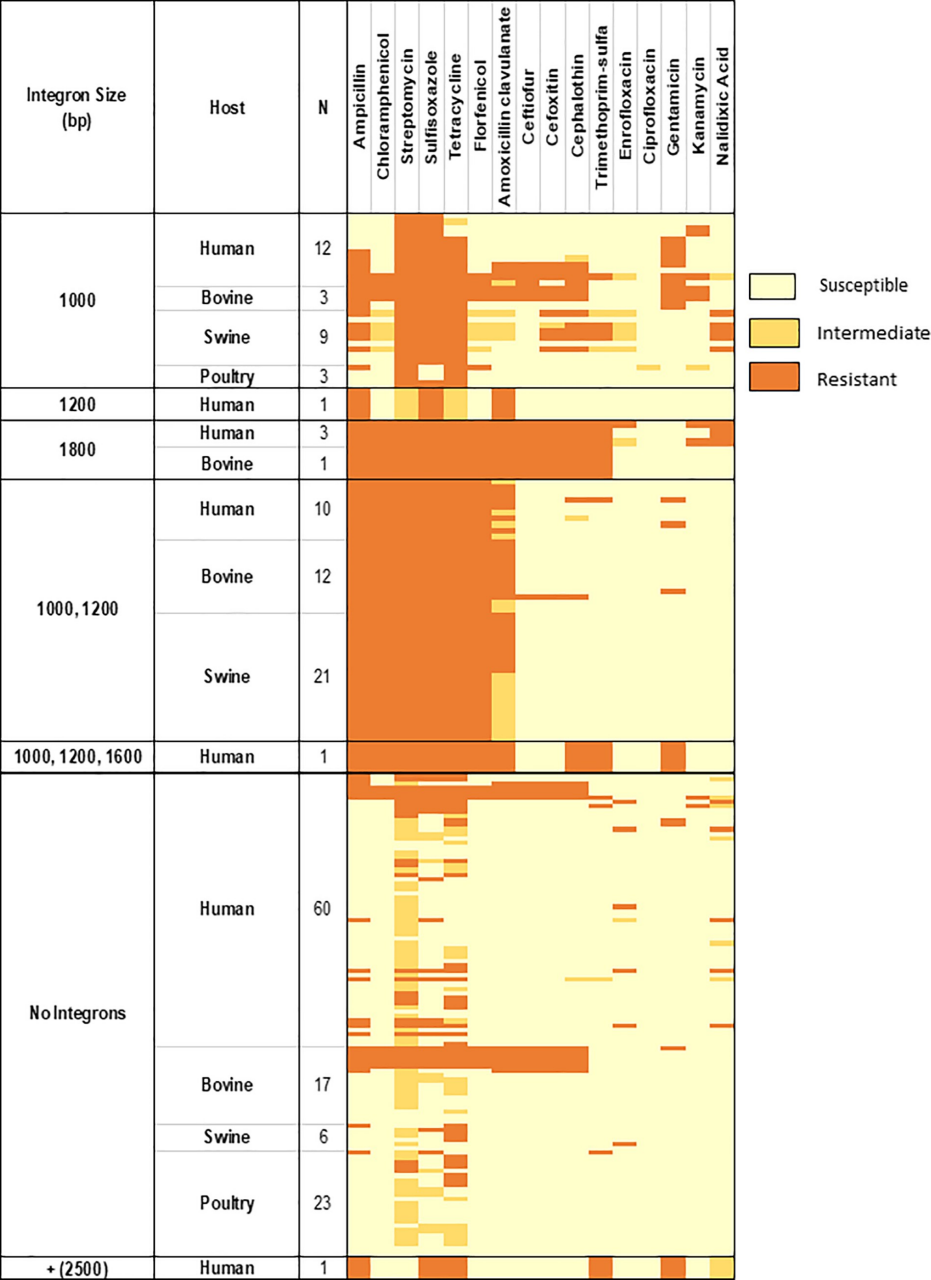
Heat map demonstrating antimicrobial susceptibility, intermediate and resistance within each integron size by host species.

The heatmap produced (Fig 2) showed AMR patterns within each integron size among various host species. There were 27 isolates (12 human, 9 swine, 3 bovine and 3 poultry) that contained only the 1000bp integron, and 100% were resistant to streptomycin, 93% were resistant to sulfisoxazole, 89% to tetracycline, and 56% to ampicillin.

Four isolates (3 human and 1 bovine) contained only an 1800bp integron, 100% of which were multi-drug resistant (MDR) to amoxicillin-clavulanic acid, ampicillin, chloramphenicol, streptomycin, sulfisoxazole, sulfa-trimethoprim, tetracycline, ceftiofur, cefoxitin, cephalothin and florfenicol. All 3 of these human isolates were also resistant to nalidixic acid and 2 of them were resistant to enrofloxacin and kanamycin. One human isolate carried a 1200bp integron with MDR to 5 antimicrobials (amoxicillin-clavulanic acid, ampicillin, streptomycin, sulfisoxazole, and tetracycline). Forty-three isolates (10 human, 21 swine and 12 bovine) contained both the 1000 and 1200bp integrons, all of which were resistant to 6 antimicrobials (ampicillin, streptomycin, sulfisoxazole, chloramphenicol, tetracycline and florfenicol (ACSSuTF)). One human isolate contained 3 integrons, 1000, 1200 and 1600bp, and was resistant to sulfa-trimethoprim, cephalothin and gentamicin along with ACSSuTF. The isolate containing +2500bp integron was resistant to ampicillin, sulfizoxazole, tetracycline, sulfa-trimethoprim, gentamicin and nalidixic acid.

One-hundred and six isolates (106/183, 57.9%), representing all host types, did not contain any class I integrons. Five bovine and 3 human isolates of those were resistant to ACSSuTF.

The presence of integron of any size was significantly associated with MDR patterns of SSuT, ACSSuT and ACSSuTF across all host species (p<0.01) except poultry (p = 0.22). The association was also significant when the data from all the host species were combined (Table 2).

**Table 2:** Association of integron presence with three MDR patterns.

Host	Integron Yes/No	Total	SSuT		Fisher's Exact/ Chi-square Test	ACSSuT		Fisher's Exact/ Chi-square Test	ACSSuTF		Fisher's Exact/ Chi-square Test
			Yes	No		Yes	No		Yes	No	
**Bovine**	**Yes**	**16**	16	0	<0.0001	15	1	0.0002	15	1	0.0002
	**No**	**17**	6	11		5	12		5	12	
	**Total**	**33**	22	11		20	13		20	13	
**Poultry**	**Yes**	**3**	1	2	0.22		3	Cannot be calculated		3	Cannot be calculated
	**No**	**23**	1	22			23			23	
	**Total**	**26**	2	24			26			26	
**Human**	**Yes**	**28**	24	4	<0.0001	16	12	<0.0001	16	12	<0.0001
	**No**	**60**	15	45		3	57		3	57	
	**Total**	**88**	39	49		19	69		19	69	
**Swine**	**Yes**	**30**	30	0	<0.0001	26	4	0.0001	26	4	0.0001
	**No**	**6**	1	5		0	6		0	6	
	**Total**	**36**	31	5		26	10		26	10	
**All hosts**	**Yes**	**77**	71	6	<0.0001	57	20	<0.0001	57	20	<0.0001
	**No**	**106**	23	83		8	98		8	98	
	**Total**	**183**	94	89		65	118		65	118	

### Integron sequence data analysis

We detected 122 integron sequences from 77 isolates, which contained a total of 127 AMR genes (Table 3).

**Table 3:** Number of isolates containing antimicrobial resistance genes compared to resistance to antimicrobial drug classes per species and integron size.

Integron size (bp)	Class	Aminoglycosides	Beta-lactams	Trimethoprim	Total
	Mechanism	Aminoglycoside O-nucleotidyltransferases	Class A beta-lactamases	Dihydrofolate reductase	
**1000**	**Bovine**	15			**15**
	**Swine**	30	1		**31**
	**Poultry**	3			**3**
	**Human**	24			**24**
	**Total**	**72**	**1**	** **	**73**
**1200**	**Bovine**		12		**12**
	**Swine**		21		**21**
	**Human**		12		**12**
	**Total**	** **	**45**	** **	**45**
**1600**	**Human**	1		1	**2**
	**Total**	1		1	**2**
**1800**	**Bovine**	1		1	**2**
	**Human**	3		2	**5**
	**Total**	**4**	** **	**3**	**7**
**Total**		**77**	**46**	**4**	**127**

A total of 127 AMR associated genes were derived from 122 integrons representing 77 isolates.

Aminoglycoside genes were *aad*A1, *aad*A2 or *aad*A3; beta-lactamase genes were *pse-1* or *carb-6*; Dihydrofolate reductase genes were *dfr*A12.

The majority (72/73, 98.6%) of the 1000bp integrons consisted of *aad*A1, *aad*A2 or *aad*A3 genes, representing resistance to the aminoglycoside class of antimicrobials. The remaining 1000bp integron, was associated with resistance to beta-lactams (in addition to the aminoglycoside class genes) and was from a swine host. One hundred percent (45/45) of the 1200bp integrons contained genes coding resistance to beta-lactams (pse-1 or carb-6) across different hosts, whereas the one (1/122) 1600bp integron, from a human-derived isolate, contained genes coding for resistance to aminoglycosides as well as trimethoprim but not beta-lactams. The 4 integrons of 1800bp size contained *aad*A1 or *aad*A2 genes, and 3 among them also contained *dfr*A12 gene, which confers resistance to trimethoprim.

**Fig 3:**
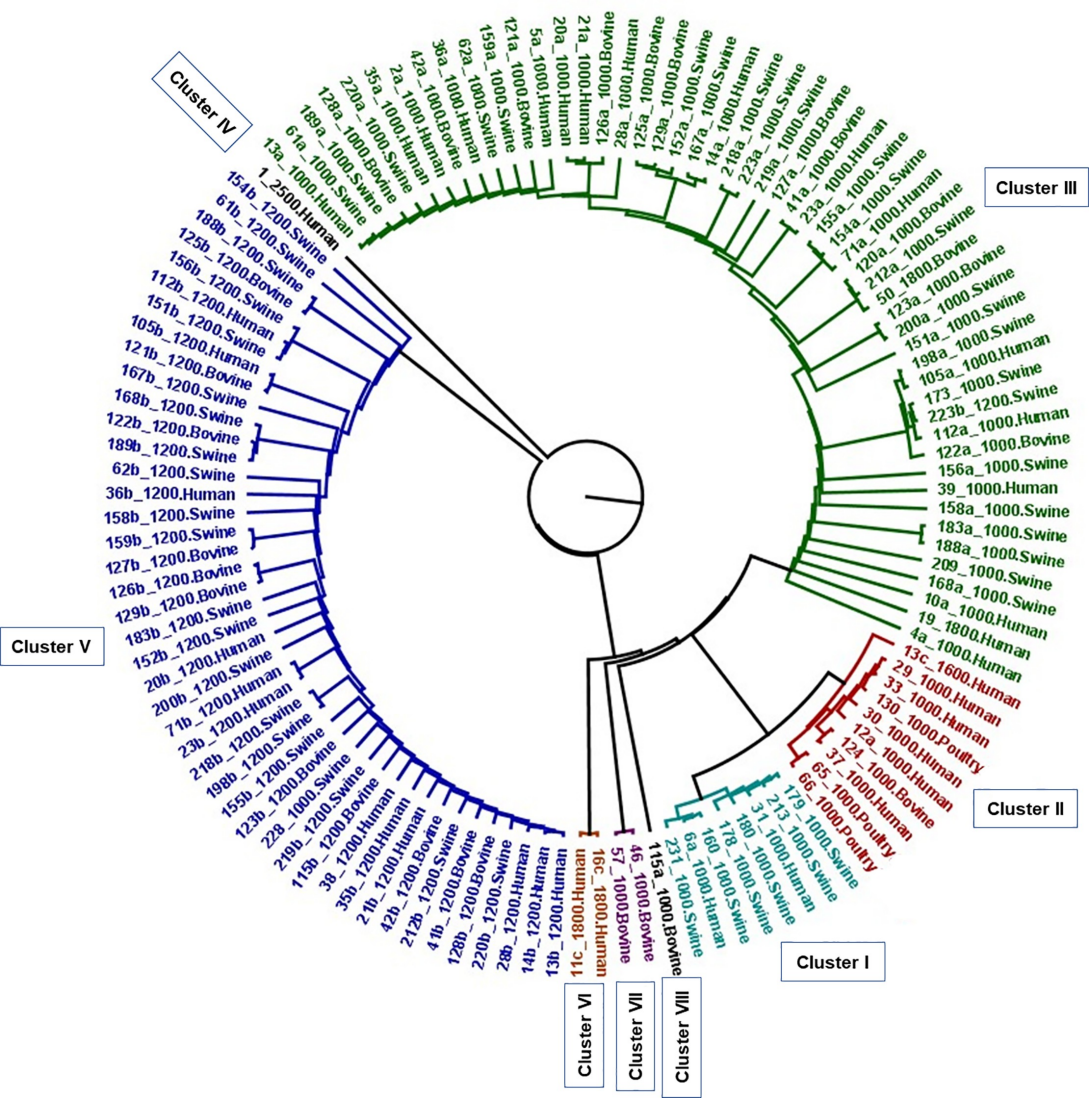
Dendrogram analysis of 121 Class I integrons obtained from *S*. Typhimurium strains from various host species.

The dendrogram that elucidated the similarity among integrons using a cut-off of 90% similarity resulted in 8 distinct clusters [Fig 3]. One of the 1200bp integrons from bovine origin was omitted from the dendrogram due to poor sequence alignment. Hence, there were 121 integrons represented in the final dendrogram. Cluster III was the largest one consisting of 43.8% (n = 53) of all integrons identified in the study, followed by cluster V consisting of 36.36% (n = 44), cluster II with 8.26% (n = 10) and cluster I with 6.61% (n = 8) of integrons. Other clusters were in small numbers of 1 or 2 integrons in each.

The largest cluster, III, contained 94% 1000bp integrons, 3.8% of 1800bp and 1.9% of 1200bp integrons. Cluster V contained 98% of 1200bp integrons and 2% (n = 1) of 1000bp integrons. Ninety percent of cluster II was represented by 1000bp integrons and the remaining (n = 1) 1600 bp integron. All of cluster I was represented by 1000bp integrons (Table 4).

**Table 4:** Number of integrons in 8 clusters stratified by integron size and host species.

Cluster	1000 bp				1200 bp			1600 bp	1800 bp		(+2500) bp
	Human	Bovine	Swine	Poultry	Human	Bovine	Swine	Human	Human	Bovine	Human
**I**	2		6								
**II**	5	1		3				1			
**III**	16	11	23	0			1		1	1	
**IV**											1
**V**			1		12	11	20				
**VI**									2		
**VII**		2									
**VIII**		1									

## Discussion

*Salmonella* Typhimurium impacts both humans and animals and is one of the most commonly reported serotypes worldwide [31,32]. Integrons are known to be a primary source of transferable resistance genes and are suspected to serve as reservoirs of AMR genes within microbial populations [33,34]. Integrons are genetic units found in many bacterial species that are defined by their ability to capture small mobile elements called gene cassettes. They contribute to the generation of AMR diversity in bacterial, plasmid, and transposon genomes and facilitate extensive sharing of genetic information among bacteria [35].

In this study, we observed AMR diversity within each host species and associations with class I integron sizes. There was a wide variety of integron sizes detected among human isolates whereas only one integron size was detected in poultry. The occurrence of integrons detected may be associated with the differences in environmental exposures and factors which the bacterial species encounters, including antimicrobial selective pressure [36]. Due to the diversity of production systems and differences in antimicrobial usage among various livestock species, the presence of AMR may vary. It should be noted that the 26 poultry isolates included in this study were from diverse sources (two institutes) and were collected over the course of 4 years, suggesting that the uniformity in integron distribution is not associated with any temporal or spatial factors. Overall, the conventional U.S. chicken industry consumes medically important antibiotics much less intensively than the conventional turkey, pig, and cattle industries [37], helping to corroborate the observation of only one integron size in the poultry isolates evaluated in this study.

Humans may be exposed to MDR pathogens through a variety of routes including environments at healthcare facilities, farm and companion animals and their food, food products made from animals, fresh produce carrying MDR pathogens acquired from contaminated soil or water, and exposure to other individuals carrying MDR microbes [1].

Numerous studies have demonstrated the significance of class I integrons and the associations with AMR, especially MDR [14,26,38,39]. Hsu et al. (2013) [38], demonstrated a significant relationship between the presence of class I integrons and AMR in different *Salmonella* serotypes from humans and various animal hosts. Additionally, a study by Lopes et al., (2016) [39] demonstrated that *S*. Typhimurium isolates from swine sampled at abattoirs carried class I integrons and exhibited MDR, underscoring the potential risk to human health when entering food chain [39].

There are five classes of integrons that have been identified [40]. Class I integrons have been found to be the major contributor to MDR in Gram-negative bacteria and play an important role in disseminating AMR genes [41]. As a way to further validate the association of a particular MDR phenotype with a particular integron presence and associated AMR genes, each identified integron sequence was further evaluated using the MEGARes database, a tool used to align input sequences against a comprehensive database of antimicrobial resistance genes. AMR genes associated with Class I integrons and identified in our study include *aad*A1, *aad*A2, *aad*A3, beta lactamase genes, and *dfr*A12. Our study demonstrated that the majority of the isolates carrying the 1000bp integrons were resistant to streptomycin, sufisoxazole and tetracyclines across all host species and carried *aad*A genes that represent resistance to aminoglycosides. The majority of samples with 1200bp integrons showed, in addition to the antibiotic resistances listed above for the 1000bp integrons, resistance to amoxicillin-clavulanate, ampicillin, chloramphenicol and florfenicol and carried beta lactamase genes.

The integron of 1600bp in one human isolate contained the *dfr*A12 gene however, the isolate conferred AMR to sulfa-trimethoprim, cephalothin and gentamicin. The 1800bp integron conferred resistance to ACSSuTF along with resistance to amoxicillin-clavulanate, sulfa-trimethoprim, ceftiofur, cefoxitin, cephalothin and carried *dfr*A12 along with *aad*A. Our results were consistent with findings of Gebreyes et al. (2004) [26] who investigated AMR and occurrence of multidrug serotypes and class I integrons among *Salmonella* from pigs. In summary, the isolates containing similar size integrons showed similar MDR patterns, with few exceptions. This was consistent across all host species evaluated in this study. Potential use of such information includes the ability to design better assays for early screening of current and emerging drug resistant *S*. Typhimurium.

## Conclusions

Our study has demonstrated a high occurrence of class I integrons of different sizes in *Salmonella* Typhimurium across various host species and their association with MDR. Only one integron size was detected in poultry isolates compared to diverse integron sizes detected among livestock species and humans. Multidrug resistant *Salmonella* is a significant public health concern and our findings point to the importance of judicious use of antimicrobials among livestock and poultry. In the future, a screening assay could be implemented after isolation of S. Typhimurium. Demonstration of 1000bp or 1000+1200bp integrons by class I integron PCR would be predictive of MDR, and assist in management and treatment decisions.

## Supporting information

S1 Fig Gel raw image. (TIF)

S1 Data (XLSX)

S2 Data (ZIP)
