# Supplementary figures and images for: Genomic diversity of class I integrons from antimicrobial resistant strains of Salmonella Typhimurium isolated from livestock, poultry and humans
Source: PLoS One. 2020 Dec 11;15(12):e0243477. doi: 10.1371/journal.pone.0243477 (PMC7732114; doi:10.1371/journal.pone.0243477)

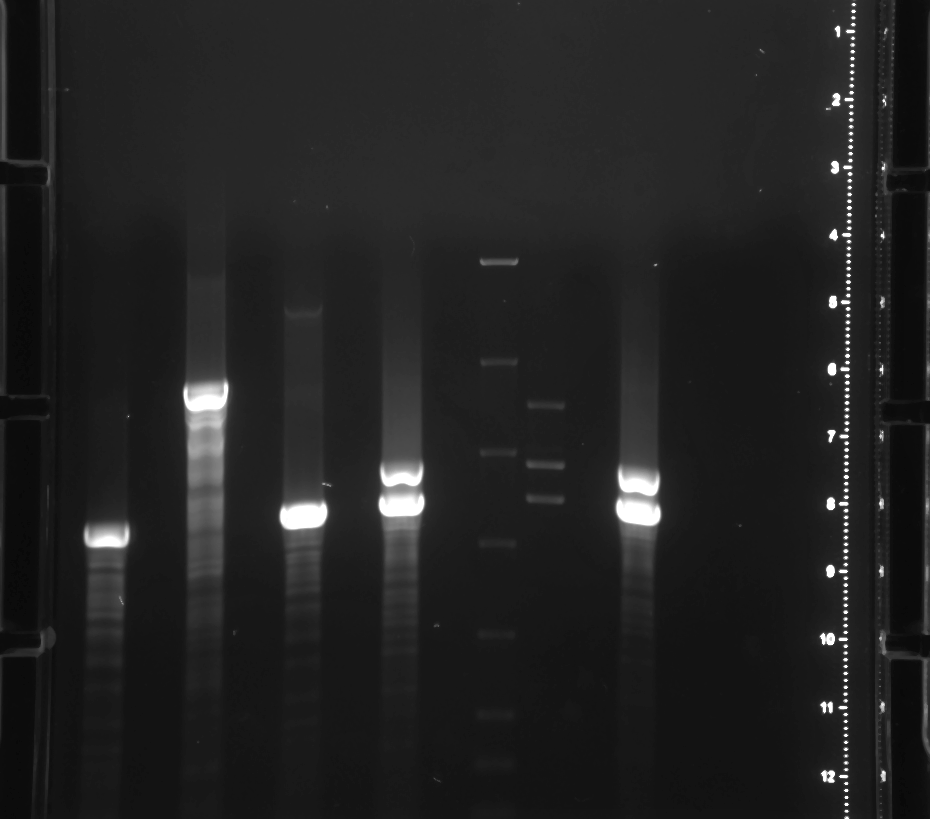

Supplement: S1 Fig — (TIF) [file pone.0243477.s001.tif]
